# Crystal structure of 1-[2-(4-chloro­phen­yl)-4,5-diphenyl-1*H*-imidazol-1-yl]propan-2-ol

**DOI:** 10.1107/S2056989016019332

**Published:** 2017-01-01

**Authors:** Shaaban K. Mohamed, Adel A. Marzouk, Mustafa R. Albayati, Antar A. Abdelhamid, Jim Simpson

**Affiliations:** a Faculty of Science and Engineering, Health Care Division, Manchester Metropolitan University, Manchester M1 5GD, England; b Chemistry Department, Faculty of Science, Minia University, 61519 El-Minia, Egypt; c Pharmaceutical Chemistry Department, Faculty of Pharmacy, Al Azhar University, 71515 Assiut, Egypt; d Kirkuk University, College of Education, Department of Chemistry, Kirkuk, Iraq; e Chemistry Department, Faculty of Science, Sohag University, Sohag, Egypt; f Department of Chemistry, University of Otago, PO Box 56, Dunedin, New Zealand

**Keywords:** crystal structure, multi-substituted imidazole, hydrogen bonding, C—H⋯π inter­actions

## Abstract

The title compound, C_24_H_21_ClN_2_O, crystallizes with two unique mol­ecules in the asymmetric unit. In each mol­ecule, the central imidazole ring is substituted at the 2-, 4- and 5-positions by benzene rings. The 2-substituted ring carries a Cl atom at the 4-position. One of the imidazole N atoms in each mol­ecule has a propan-2-ol substituent. In the crystal, a series of O—H⋯N, C—H⋯O and C—H⋯Cl hydrogen bonds, augmented by several C—H⋯π(ring) inter­actions, generate a three-dimensional network of mol­ecules stacked along the *a*-axis direction.

## Abstract 2

The mol­ecular and crystal structure of the title imidazole derivative is reported. The structure is stabilized by an extensive O—H⋯N, C—H⋯O/Cl and C—H⋯π(ring) hydrogen-bonding network.

## Chemical context   

Imidazole derivatives are important components of numerous natural products and are especially noted for their numerous pharmacological applications, particularly as anti-tumour agents (Bahnous *et al.*, 2013 ▸; Belwal & Joshi, 2012 ▸). In addition, they also display anti-bacterial fungicidal and anti-parasitic properties (Sridharan *et al.*, 2014 ▸; Mohammadi *et al.*, 2012 ▸; Sharma *et al.*, 2009 ▸). We have recently developed fast and efficient multi-component reactions, catalysed by the ionic liquid morphilinium hydrogen sulfate, to prepare imidazole derivatives in a single-step process (Marzouk *et al.*, 2016 ▸). The title compound is the result of just such a synthetic process and we report its crystal structure here.
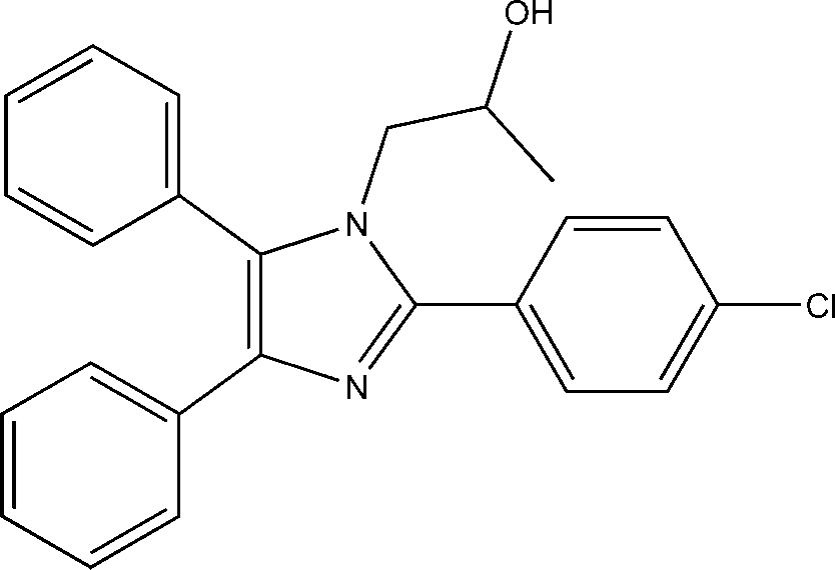


## Structural commentary   

**Figure 1:**
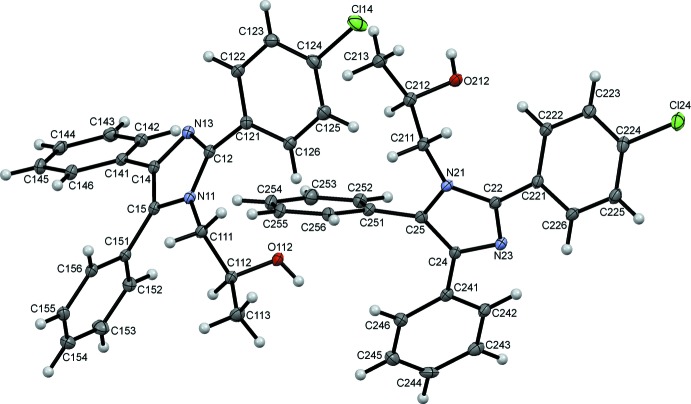
The asymmetric unit of (I), with displacement ellipsoids drawn at the 50% probability level.

**Figure 2:**
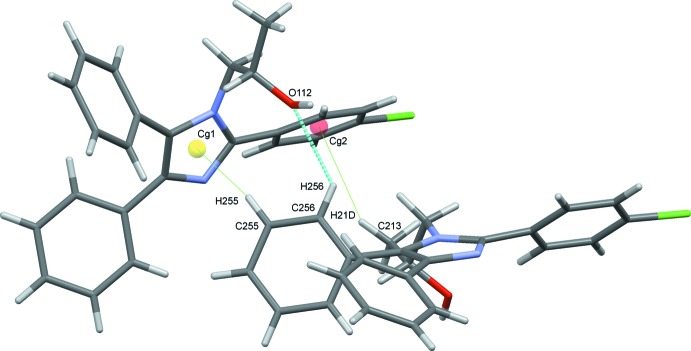
C—H⋯O (dashed blue lines) and C—H⋯π hydrogen bonds (dotted green lines) link the unique mol­ecules in the asymmetric unit of (I).

**Figure 3:**
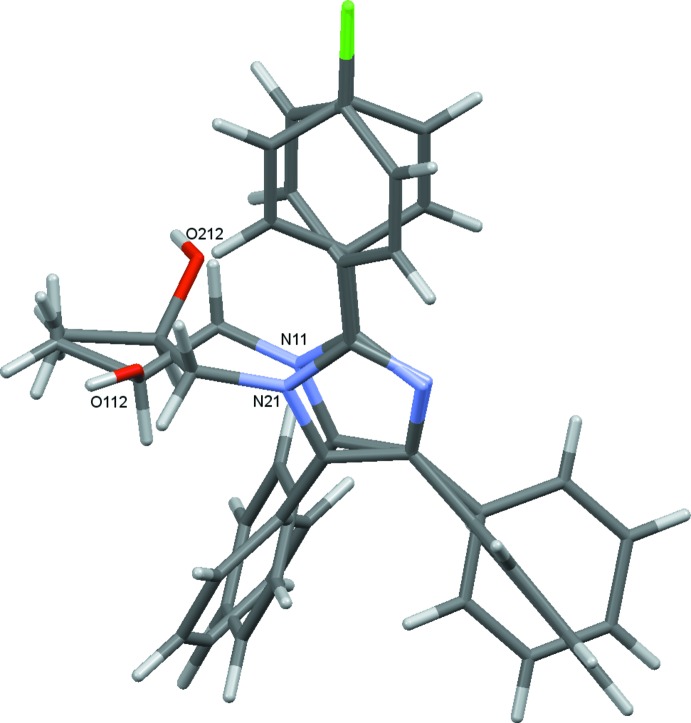
An overlay (Macrae *et al.*, 2008 ▸) of the two mol­ecules.

The title compound, (I), crystallizes with two unique mol­ecules, 1 and 2, in the asymmetric unit, differentiated by the leading digits 1 and 2 in the numbering scheme, Fig. 1 ▸. The two mol­ecules are linked in the asymmetric unit by a C256—H256⋯O12 hydrogen bond augmented by two C—H⋯π(ring) contacts, C213—H21*D*⋯*Cg*2 and C255—H255⋯*Cg*1 (Fig. 2 ▸ and Table 1 ▸). Each mol­ecule consists of a central imidazole ring substituted at the 2-, 4- and 5-positions with benzene rings. The 2-phenyl substituents carry chlorine atoms at the 4-position. The N11 and N21 atoms have propan-2-ol substituents. The benzene rings of the two unique mol­ecules subtend dihedral angles of 40.83 (12) and 39.01 (14)° to C121–C126 and C221–C226, 43.34 (13) and 34.80 (15)° to C141–C146 and C241–C246 and 59.91 (11) and 63.53 (11)° to C151–C156 and C251–C256, respectively. The approximately planar N11/C111–C113 and N21/C211– C213 propane chains (r.m.s. deviations of 0.0413 and 0.0431 Å, respectively) are inclined to the imidazole rings by 74.25 (16) and 72.94 (15)°. Bond distances and angles in the imidazole rings and their propanol substituents are reasonably similar for the two unique mol­ecules and are also similar to those in the archetypal lophine, 2,4,5-triphenyl-1*H*-imidazole (Yanover & Kaftory, 2009 ▸), and the closely related 2-(2,4,5-triphenyl-1*H*-imidazol-1-yl)ethanol (Mohamed *et al.*, 2015 ▸). However, an overlay, Fig. 3 ▸ (Macrae *et al.*, 2008 ▸), reveals an r.m.s. deviation of 1.189 Å, largely due to the considerable variation in the orientations of the benzene rings between the two mol­ecules.

## Supra­molecular features   

**Figure 4:**
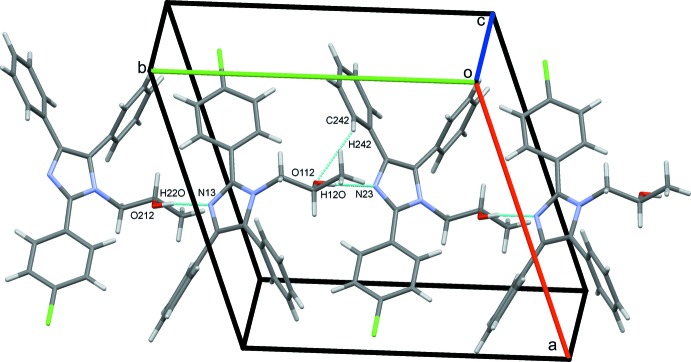
O—H⋯N and C—H⋯O hydrogen bonds form zigzag *C*(7) chains of type 1 and 2 mol­ecules along *b*.

**Figure 5:**
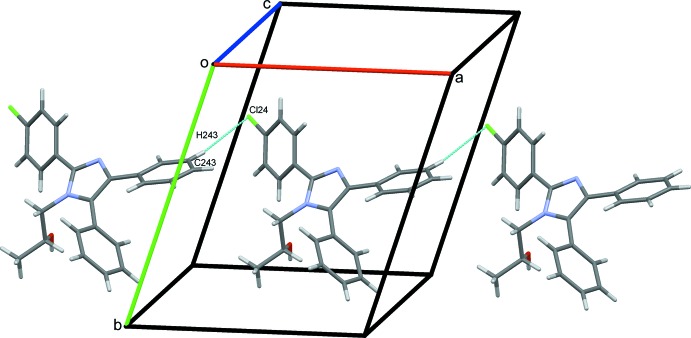
*C*(12) chains of type 2 mol­ecules along *a* formed by C—H⋯Cl hydrogen bonds.

**Figure 6:**
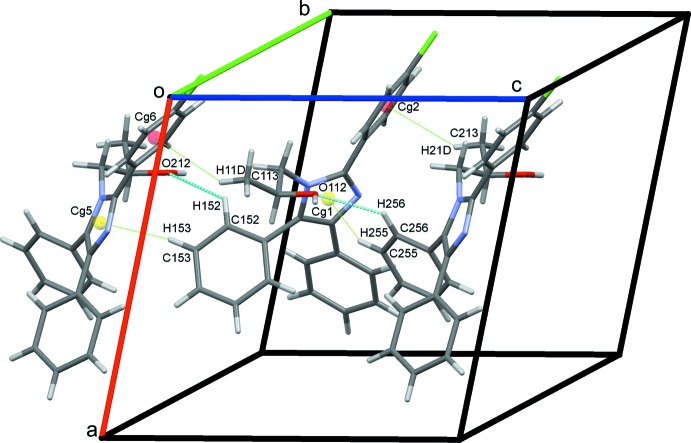
Rows of type 1 and 2 mol­ecules along *c* linked by C—H⋯π hydrogen bonds.

**Figure 7:**
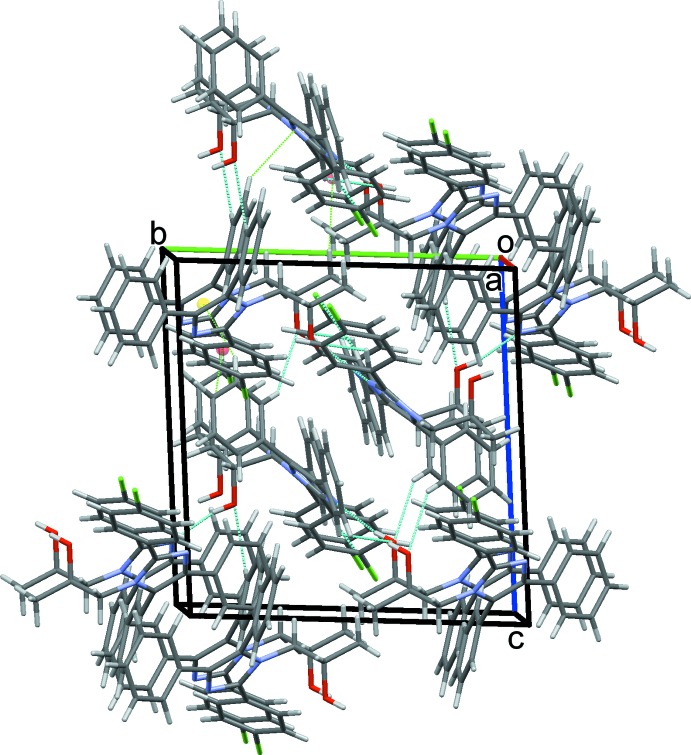
The overall packing of the two mol­ecules of (I), viewed along the *a* axis.

O112–H12*O*⋯N23 hydrogen bonds supported by C242—H242⋯O112 contacts combine with O212—H22*O*⋯N13 hydrogen bonds to link alternate type 1 and 2 mol­ecules in a head-to-tail fashion, forming *C*(7) chains along *b*, Fig. 4 ▸. C243—H243⋯Cl24 hydrogen bonds link adjacent type 2 mol­ecules into *C*(12) chains along the *a-*axis direction, Fig. 5 ▸. C—H⋯π contacts also play a role in establishing the packing, although no π–π stacking inter­actions are observed, despite the abundance of aromatic rings. Hence C153—H153⋯*Cg*5 and C255—H255⋯*Cg*1 contacts combine with C113—H11*D*⋯*Cg*6, C213—H21*D*⋯*Cg*2 and two C—H⋯O hydrogen bonds, Table 1 ▸, to form head-to-head chains of alternating type 1 and type 2 mol­ecules along the *c* axis, Fig. 6 ▸. An inter­esting feature of the packing of these mol­ecules is the formation of significant voids in the crystal structure with a volume amounting to 2039 Å^3^ across the unit cell. This large void is unexpected as no solvent appeared and the final difference map was reasonably flat (see _refine_special_details in the CIF). The mol­ecules stack in an orderly fashion along each of the three principal crystallographic axes and the voids are clearly visible in views of the overall packing along these directions, see for example Fig. 7 ▸.

## Database survey   

A search of the Cambridge Structural Database (Version 5.37 with two updates; Groom *et al.*, 2016 ▸) for an imidazole ring with phenyl substituents at the 4- and 5- positions, a methyl­ene group at N1 and a benzene ring at C2 yielded 33 hits with the closest matches to the title compound being the related alcohol derivatives 4-[1-(2-hy­droxy­prop­yl)-4,5-diphenyl-1*H*-imidazol-2-yl]benzoic acid (Jasinski *et al.*, 2015 ▸), 1-[2-(2,6-dichloro­phen­yl)-4,5-diphenyl-1*H*-imidazol-1-yl]propan-2-ol (XULMEY; Akkurt *et al.*, 2015 ▸), 2-[2-(4-meth­oxy­phen­yl)-4,5-diphenyl-1*H*-imidazol-1-yl]ethanol (WIHHOM; Mohamed *et al.*, 2013*a* ▸) and three others with ethanol substituents on N1, VUWGAX, VUWGEB, VUWGIF (Mohamed *et al.*, 2015 ▸). Inter­estingly, five unique structures [AFUVUU (Mohamed *et al.*, 2013*b* ▸), IFUMON (Mohamed *et al.*, 2013*c* ▸), OZEGEG (Kapoor *et al.*, 2011 ▸), YOCTAM (Ghoranneviss *et al.*, 2008 ▸) and SUYZIX (Rajaraman *et al.*, 2016 ▸)] are found of related compounds with 4-chloro­phenyl groups on C2 and but none of these have alcohol substituents on N1.

## Synthesis and crystallization   

The compound was prepared by a literature procedure (Marzouk *et al.*, 2016 ▸). Irregular colourless block-like crystals were grown from ethanol solution at room temperature.

## Refinement   

Crystal data, data collection and structure refinement details are summarized in Table 2 ▸. The hydrogen atoms on O112 and O212 were located in a difference Fourier map and their coordinates refined with *U*_iso_ = 1.5 *U*_eq_ (O). All other H atoms were refined using a riding model with *d*(C—H) = 0.95 Å, *U*_iso_ = 1.2*U*_eq_(C) for aromatic, 1.00 Å for methine and 0.99 Å for CH_2_ H atoms, all with *U*_iso_ = 1.2*U*_eq_(C) and 0.98 Å, *U*_iso_ = 1.5*U*_eq_(C) for CH_3_ H atoms. Seven reflections with *F*_o_ >>> *F*_c_, were omitted from the final refinement cycles.

## Supplementary Material

Crystal structure: contains datablock(s) global, I. DOI: 10.1107/S2056989016019332/hg5480sup1.cif

Structure factors: contains datablock(s) I. DOI: 10.1107/S2056989016019332/hg5480Isup2.hkl

Supporting information file. DOI: 10.1107/S2056989016019332/hg5480Isup3.cml

CCDC reference: 1520559

Additional supporting information: crystallographic information; 3D view; checkCIF report

## Figures and Tables

**Table 1:** Hydrogen-bond geometry (Å, °) *Cg*1, *Cg*2, *Cg*5 and *Cg*6 are the centroids of the N11/C12/N13/C14/C15, N21/C22/N23/C24/C25, C221–C226 and C241–C246 rings, respectively.

*D*—H⋯*A*	*D*—H	H⋯*A*	*D*⋯*A*	*D*—H⋯*A*
O112—H12*O*⋯N23^i^	0.80 (5)	2.01 (5)	2.804 (3)	170 (4)
O212—H22*O*⋯N13^ii^	0.82 (5)	1.99 (5)	2.790 (3)	165 (4)
C152—H152⋯O212^iii^	0.95	2.61	3.227 (4)	123
C256—H256⋯O112	0.95	2.48	3.162 (4)	129
C242—H242⋯O112^i^	0.95	2.68	3.277 (4)	122
C243—H243⋯Cl24^iv^	0.95	2.91	3.836 (3)	166
C113—H11*D*⋯*Cg*6^iii^	0.98	2.96	3.778 (3)	142
C153—H153⋯*Cg*5^iii^	0.95	2.65	3.495 (3)	148
C213—H21*D*⋯*Cg*2	0.98	2.91	3.745 (4)	144
C255—H255⋯*Cg*1	0.95	2.65	3.505 (3)	149

Symmetry codes: (i) 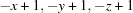; (ii) 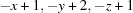; (iii) 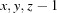; (iv) 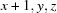.

**Table 2:** Experimental details

Crystal data	
Chemical formula	C_24_H_21_ClN_2_O
*M*_r_	388.88
Crystal system, space group	Triclinic, *P* 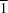
Temperature (K)	100
*a*, *b*, *c* (Å)	12.0235 (8), 13.4263 (7), 13.6588 (4)
α, β, γ (°)	90.297 (3), 98.481 (4), 110.480 (5)
*V* (Å^3^)	2039.16 (19)
*Z*	4
Radiation type	Cu *K*α
μ (mm^−1^)	1.78
Crystal size (mm)	0.26 × 0.17 × 0.12

Data collection	
Diffractometer	Agilent SuperNova Dual Source diffractometer with an Atlas detector
Absorption correction	Gaussian (*CrysAlis PRO*; Agilent, 2014 ▸)
*T*_min_, *T*_max_	0.929, 0.958
No. of measured, independent and observed [*I* > 2σ(*I*)] reflections	20056, 8427, 7077
*R*_int_	0.046
(sin θ/λ)_max_ (Å^−1^)	0.631

Refinement	
*R*[*F* ^2^ > 2σ(*F* ^2^)], *wR*(*F* ^2^), *S*	0.071, 0.188, 1.09
No. of reflections	8427
No. of parameters	513
H-atom treatment	H atoms treated by a mixture of independent and constrained refinement
Δρ_max_, Δρ_min_ (e Å^−3^)	0.61, −0.38

Computer programs: *CrysAlis PRO* (Agilent (2014 ▸), *SHELXS2013* (Sheldrick, 2008 ▸), *SHELXL2014* (Sheldrick, 2015 ▸), *TITAN2000* (Hunter & Simpson, 1999 ▸), *Mercury* (Macrae *et al.*, 2008 ▸), *enCIFer* (Allen *et al.*, 2004 ▸), *PLATON* (Spek, 2009 ▸), *publCIF* (Westrip 2010 ▸) and *WinGX* (Farrugia 2012 ▸).

## supplementary crystallographic information

### Crystal data

**Table:**

C_24_H_21_ClN_2_O	*Z* = 4
*M**_r_* = 388.88	*F*(000) = 816
Triclinic, *P*1	*D*_x_ = 1.267 Mg m^−^^3^
*a* = 12.0235 (8) Å	Cu *K*α radiation, λ = 1.54184 Å
*b* = 13.4263 (7) Å	Cell parameters from 8456 reflections
*c* = 13.6588 (4) Å	θ = 3.3–76.0°
α = 90.297 (3)°	µ = 1.78 mm^−^^1^
β = 98.481 (4)°	*T* = 100 K
γ = 110.480 (5)°	Block, colourless
*V* = 2039.16 (19) Å^3^	0.26 × 0.17 × 0.12 mm

### Data collection

**Table:**

Agilent SuperNova Dual Source diffractometer with an Atlas detector	8427 independent reflections
Radiation source: sealed X-ray tube, SuperNova (Cu) X-ray Source	7077 reflections with *I* > 2σ(*I*)
Mirror monochromator	*R*_int_ = 0.046
Detector resolution: 10.3449 pixels mm^-1^	θ_max_ = 76.5°, θ_min_ = 3.3°
ω scans	*h* = −14→15
Absorption correction: gaussian (CrysAlis PRO; Agilent, 2014)	*k* = −16→16
*T*_min_ = 0.929, *T*_max_ = 0.958	*l* = −16→17
20056 measured reflections	

### Refinement

**Table:**

Refinement on *F*^2^	0 restraints
Least-squares matrix: full	Hydrogen site location: mixed
*R*[*F*^2^ > 2σ(*F*^2^)] = 0.071	H atoms treated by a mixture of independent and constrained refinement
*wR*(*F*^2^) = 0.188	*w* = 1/[σ^2^(*F*_o_^2^) + (0.0644*P*)^2^ + 4.0157*P*] where *P* = (*F*_o_^2^ + 2*F*_c_^2^)/3
*S* = 1.09	(Δ/σ)_max_ < 0.001
8427 reflections	Δρ_max_ = 0.61 e Å^−^^3^
513 parameters	Δρ_min_ = −0.38 e Å^−^^3^

### Special details

**Table:**

Geometry. All esds (except the esd in the dihedral angle between two l.s. planes) are estimated using the full covariance matrix. The cell esds are taken into account individually in the estimation of esds in distances, angles and torsion angles; correlations between esds in cell parameters are only used when they are defined by crystal symmetry. An approximate (isotropic) treatment of cell esds is used for estimating esds involving l.s. planes.
Refinement. 7 reflections with Fo >>> Fc were omitted from the final refinement cycles.The large void volume is unexpected as no solvent appeared or has been SQUEEZED out. The final difference map was reasonably flat; see below:Electron density synthesis with coefficients Fo-FcHighest peak 0.61 at 0.0329 0.0214 0.3946 [ 2.26 A from H152 ] Deepest hole -0.39 at 0.0342 0.2000 0.1384 [ 0.55 A from CL14 ]Mean = 0.00, Rms deviation from mean = 0.08, Highest memory used = 7053 / 34831Fourier peaks appended to .res file x y z sof U Peak Distances to nearest atoms (including eq.)Q1 1 0.9671 0.9786 0.6054 1.00000 0.05 0.61 2.26 H152 2.55 H153 2.68 C152 2.83 C153 Q2 1 1.0187 1.1772 0.8923 1.00000 0.05 0.55 2.13 H143 2.54 H252 2.60 H253 2.79 C143 Q3 1 0.9545 0.5837 1.0817 1.00000 0.05 0.40 0.69 C244 0.98 C245 1.18 H244 1.48 H245 Q4 1 0.5215 0.9173 1.1876 1.00000 0.05 0.40 1.03 H22O 1.29 O212 1.55 H212 1.65 C212 Q5 1 0.4633 1.0258 1.1059 1.00000 0.05 0.39 1.05 H21E 1.93 C213 2.36 H22O 2.38 H21D Q6 1 0.4358 0.4634 1.1908 1.00000 0.05 0.38 1.24 N23 1.47 H226 1.55 C22 1.83 H12O Q7 1 0.4296 0.9995 0.7074 1.00000 0.05 0.37 1.27 H122 1.39 N13 1.69 C12 1.70 C122 Q8 1 0.5533 0.7461 0.3069 1.00000 0.05 0.36 0.79 H155 1.07 C155 1.57 C156 1.69 H156 Q9 1 0.5676 0.7949 0.7972 1.00000 0.05 0.35 0.73 H255 1.16 C255 1.69 C256 1.77 H256 Q10 1 0.4633 0.4418 0.6084 1.00000 0.05 0.35 0.95 H11C 1.87 C113 2.32 H11D 2.32 H11DShortest distances between peaks (including symmetry equivalents) 4 7 1.71 4 5 2.09 8 10 2.74 5 7 2.76 5 9 2.90 6 10 2.92 4 8 2.93

### Fractional atomic coordinates and isotropic or equivalent isotropic displacement parameters (Å^2^)

**Table:**

	*x*	*y*	*z*	*U*_iso_*/*U*_eq_	
N11	0.4623 (2)	0.81917 (18)	0.10692 (17)	0.0206 (5)	
C111	0.3879 (3)	0.7109 (2)	0.0663 (2)	0.0217 (6)	
H11A	0.3799	0.7087	−0.0069	0.026*	
H11B	0.3065	0.6925	0.0840	0.026*	
C112	0.4415 (3)	0.6284 (2)	0.1053 (2)	0.0229 (6)	
H112	0.5267	0.6510	0.0934	0.027*	
O112	0.4398 (2)	0.62826 (17)	0.20851 (15)	0.0244 (4)	
H12O	0.450 (4)	0.577 (4)	0.233 (3)	0.037*	
C113	0.3704 (3)	0.5195 (2)	0.0524 (2)	0.0306 (7)	
H11C	0.4005	0.4661	0.0832	0.046*	
H11D	0.3800	0.5220	−0.0177	0.046*	
H11E	0.2851	0.5004	0.0576	0.046*	
C12	0.4413 (3)	0.8817 (2)	0.1760 (2)	0.0204 (5)	
C121	0.3326 (3)	0.8574 (2)	0.2233 (2)	0.0225 (6)	
C122	0.2886 (3)	0.9401 (2)	0.2337 (2)	0.0254 (6)	
H122	0.3273	1.0071	0.2084	0.030*	
C123	0.1899 (3)	0.9258 (3)	0.2803 (2)	0.0294 (6)	
H123	0.1604	0.9821	0.2865	0.035*	
C124	0.1348 (3)	0.8281 (3)	0.3175 (2)	0.0297 (7)	
Cl14	0.01324 (8)	0.81097 (7)	0.37944 (7)	0.0422 (2)	
C125	0.1763 (3)	0.7448 (2)	0.3090 (2)	0.0303 (7)	
H125	0.1376	0.6783	0.3351	0.036*	
C126	0.2752 (3)	0.7594 (2)	0.2616 (2)	0.0252 (6)	
H126	0.3040	0.7026	0.2552	0.030*	
N13	0.5298 (2)	0.97600 (19)	0.19205 (18)	0.0221 (5)	
C14	0.6116 (3)	0.9743 (2)	0.1323 (2)	0.0215 (6)	
C141	0.7228 (3)	1.0659 (2)	0.1281 (2)	0.0219 (6)	
C142	0.7583 (3)	1.0951 (2)	0.0365 (2)	0.0258 (6)	
H142	0.7095	1.0569	−0.0225	0.031*	
C143	0.8643 (3)	1.1796 (2)	0.0304 (2)	0.0291 (6)	
H143	0.8868	1.1994	−0.0325	0.035*	
C144	0.9373 (3)	1.2353 (2)	0.1166 (3)	0.0295 (7)	
H144	1.0106	1.2921	0.1130	0.035*	
C145	0.9017 (3)	1.2068 (2)	0.2084 (2)	0.0287 (6)	
H145	0.9507	1.2449	0.2675	0.034*	
C146	0.7951 (3)	1.1232 (2)	0.2139 (2)	0.0261 (6)	
H146	0.7712	1.1048	0.2767	0.031*	
C15	0.5722 (3)	0.8776 (2)	0.0793 (2)	0.0208 (5)	
C151	0.6323 (3)	0.8381 (2)	0.0091 (2)	0.0216 (6)	
C152	0.5807 (3)	0.8094 (2)	−0.0905 (2)	0.0233 (6)	
H152	0.5031	0.8113	−0.1141	0.028*	
C153	0.6438 (3)	0.7782 (2)	−0.1547 (2)	0.0268 (6)	
H153	0.6087	0.7584	−0.2222	0.032*	
C154	0.7570 (3)	0.7758 (3)	−0.1214 (3)	0.0317 (7)	
H154	0.7992	0.7541	−0.1658	0.038*	
C155	0.8089 (3)	0.8048 (3)	−0.0231 (3)	0.0335 (7)	
H155	0.8869	0.8035	−0.0004	0.040*	
C156	0.7470 (3)	0.8359 (3)	0.0423 (2)	0.0269 (6)	
H156	0.7828	0.8557	0.1097	0.032*	
N21	0.4612 (2)	0.65591 (18)	0.60387 (18)	0.0214 (5)	
C211	0.3840 (3)	0.7139 (2)	0.5615 (2)	0.0234 (6)	
H21A	0.3028	0.6797	0.5792	0.028*	
H21B	0.3761	0.7086	0.4883	0.028*	
C212	0.4327 (3)	0.8315 (2)	0.5977 (2)	0.0267 (6)	
H212	0.5178	0.8643	0.5859	0.032*	
O212	0.4308 (2)	0.83421 (17)	0.70054 (16)	0.0261 (4)	
H22O	0.448 (4)	0.895 (4)	0.723 (3)	0.039*	
C213	0.3580 (4)	0.8907 (3)	0.5418 (3)	0.0343 (7)	
H21C	0.2734	0.8558	0.5490	0.051*	
H21D	0.3658	0.8898	0.4714	0.051*	
H21E	0.3868	0.9646	0.5691	0.051*	
C22	0.4392 (3)	0.5779 (2)	0.6698 (2)	0.0209 (5)	
C221	0.3353 (3)	0.5378 (2)	0.7228 (2)	0.0226 (6)	
C222	0.2854 (3)	0.6057 (2)	0.7629 (2)	0.0233 (6)	
H222	0.3134	0.6794	0.7510	0.028*	
C223	0.1948 (3)	0.5652 (2)	0.8201 (2)	0.0260 (6)	
H223	0.1613	0.6112	0.8477	0.031*	
C224	0.1539 (3)	0.4582 (3)	0.8365 (2)	0.0278 (6)	
Cl24	0.04309 (7)	0.40905 (7)	0.91146 (6)	0.0352 (2)	
C225	0.2008 (3)	0.3891 (2)	0.7968 (2)	0.0279 (6)	
H225	0.1714	0.3154	0.8082	0.034*	
C226	0.2911 (3)	0.4297 (2)	0.7401 (2)	0.0257 (6)	
H226	0.3238	0.3830	0.7125	0.031*	
N23	0.5282 (2)	0.53968 (19)	0.68408 (18)	0.0223 (5)	
C24	0.6107 (3)	0.5952 (2)	0.6257 (2)	0.0218 (6)	
C241	0.7235 (3)	0.5762 (2)	0.6227 (2)	0.0243 (6)	
C242	0.7849 (3)	0.5493 (2)	0.7075 (2)	0.0254 (6)	
H242	0.7522	0.5411	0.7674	0.031*	
C243	0.8928 (3)	0.5345 (2)	0.7052 (3)	0.0296 (7)	
H243	0.9330	0.5154	0.7632	0.036*	
C244	0.9431 (3)	0.5475 (3)	0.6176 (3)	0.0327 (7)	
H244	1.0184	0.5396	0.6165	0.039*	
C245	0.8820 (3)	0.5719 (3)	0.5334 (3)	0.0356 (8)	
H245	0.9147	0.5794	0.4734	0.043*	
C246	0.7726 (3)	0.5858 (3)	0.5351 (2)	0.0295 (7)	
H246	0.7310	0.6019	0.4762	0.035*	
C25	0.5715 (3)	0.6682 (2)	0.5762 (2)	0.0218 (6)	
C251	0.6349 (3)	0.7504 (2)	0.5120 (2)	0.0229 (6)	
C252	0.7451 (3)	0.8269 (2)	0.5516 (2)	0.0276 (6)	
H252	0.7752	0.8286	0.6201	0.033*	
C253	0.8116 (3)	0.9009 (3)	0.4924 (3)	0.0342 (7)	
H253	0.8868	0.9528	0.5202	0.041*	
C254	0.7675 (3)	0.8988 (3)	0.3917 (3)	0.0341 (7)	
H254	0.8130	0.9488	0.3506	0.041*	
C255	0.6571 (3)	0.8234 (3)	0.3518 (2)	0.0307 (7)	
H255	0.6269	0.8224	0.2834	0.037*	
C256	0.5905 (3)	0.7494 (2)	0.4111 (2)	0.0263 (6)	
H256	0.5149	0.6982	0.3833	0.032*	

### Atomic displacement parameters (Å^2^)

**Table:**

	*U*^11^	*U*^22^	*U*^33^	*U*^12^	*U*^13^	*U*^23^
N11	0.0264 (12)	0.0161 (11)	0.0195 (11)	0.0071 (9)	0.0054 (9)	0.0044 (9)
C111	0.0261 (14)	0.0165 (12)	0.0216 (13)	0.0065 (11)	0.0038 (11)	0.0033 (10)
C112	0.0298 (15)	0.0190 (13)	0.0211 (14)	0.0095 (11)	0.0060 (11)	0.0047 (11)
O112	0.0364 (12)	0.0204 (10)	0.0198 (10)	0.0138 (9)	0.0052 (8)	0.0065 (8)
C113	0.0407 (18)	0.0206 (14)	0.0297 (16)	0.0099 (13)	0.0058 (13)	0.0019 (12)
C12	0.0253 (14)	0.0176 (12)	0.0197 (13)	0.0085 (11)	0.0059 (10)	0.0063 (10)
C121	0.0270 (14)	0.0218 (13)	0.0199 (13)	0.0088 (11)	0.0066 (11)	0.0036 (11)
C122	0.0339 (16)	0.0181 (13)	0.0263 (15)	0.0101 (12)	0.0088 (12)	0.0045 (11)
C123	0.0307 (16)	0.0251 (15)	0.0355 (17)	0.0120 (12)	0.0099 (13)	0.0005 (13)
C124	0.0296 (16)	0.0277 (15)	0.0316 (16)	0.0064 (12)	0.0137 (13)	−0.0014 (12)
Cl14	0.0359 (4)	0.0339 (4)	0.0567 (6)	0.0050 (3)	0.0257 (4)	−0.0047 (4)
C125	0.0360 (17)	0.0204 (14)	0.0335 (17)	0.0057 (12)	0.0126 (13)	0.0024 (12)
C126	0.0301 (15)	0.0190 (13)	0.0276 (15)	0.0081 (11)	0.0093 (12)	0.0037 (11)
N13	0.0290 (13)	0.0195 (11)	0.0202 (11)	0.0101 (10)	0.0075 (9)	0.0041 (9)
C14	0.0291 (15)	0.0195 (13)	0.0183 (13)	0.0104 (11)	0.0065 (11)	0.0063 (10)
C141	0.0265 (14)	0.0184 (13)	0.0243 (14)	0.0110 (11)	0.0077 (11)	0.0065 (11)
C142	0.0308 (15)	0.0227 (14)	0.0254 (15)	0.0098 (12)	0.0075 (12)	0.0081 (11)
C143	0.0339 (16)	0.0244 (14)	0.0318 (16)	0.0098 (13)	0.0148 (13)	0.0110 (12)
C144	0.0304 (16)	0.0174 (13)	0.0411 (18)	0.0069 (12)	0.0106 (13)	0.0074 (12)
C145	0.0312 (16)	0.0212 (14)	0.0334 (17)	0.0087 (12)	0.0056 (13)	0.0009 (12)
C146	0.0350 (16)	0.0220 (14)	0.0236 (14)	0.0115 (12)	0.0078 (12)	0.0038 (11)
C15	0.0251 (14)	0.0186 (13)	0.0204 (13)	0.0087 (11)	0.0065 (11)	0.0065 (10)
C151	0.0292 (14)	0.0155 (12)	0.0223 (14)	0.0088 (11)	0.0093 (11)	0.0056 (10)
C152	0.0300 (15)	0.0187 (13)	0.0228 (14)	0.0090 (11)	0.0077 (11)	0.0058 (11)
C153	0.0378 (17)	0.0218 (14)	0.0212 (14)	0.0094 (12)	0.0090 (12)	0.0027 (11)
C154	0.0410 (18)	0.0295 (16)	0.0319 (17)	0.0173 (14)	0.0156 (14)	0.0032 (13)
C155	0.0331 (17)	0.0410 (18)	0.0330 (17)	0.0203 (15)	0.0075 (13)	0.0014 (14)
C156	0.0312 (16)	0.0299 (15)	0.0229 (14)	0.0143 (13)	0.0058 (12)	0.0046 (12)
N21	0.0289 (13)	0.0165 (11)	0.0205 (11)	0.0101 (9)	0.0043 (9)	0.0034 (9)
C211	0.0296 (15)	0.0228 (14)	0.0202 (13)	0.0130 (12)	0.0025 (11)	0.0052 (11)
C212	0.0361 (16)	0.0235 (14)	0.0238 (15)	0.0136 (12)	0.0076 (12)	0.0063 (11)
O212	0.0387 (12)	0.0171 (10)	0.0235 (10)	0.0103 (9)	0.0067 (9)	0.0032 (8)
C213	0.051 (2)	0.0293 (16)	0.0306 (17)	0.0239 (15)	0.0081 (15)	0.0063 (13)
C22	0.0269 (14)	0.0157 (12)	0.0193 (13)	0.0070 (11)	0.0030 (11)	0.0016 (10)
C221	0.0281 (14)	0.0202 (13)	0.0196 (13)	0.0085 (11)	0.0039 (11)	0.0062 (10)
C222	0.0286 (15)	0.0198 (13)	0.0233 (14)	0.0105 (11)	0.0050 (11)	0.0059 (11)
C223	0.0282 (15)	0.0269 (15)	0.0260 (15)	0.0130 (12)	0.0059 (12)	0.0072 (12)
C224	0.0273 (15)	0.0289 (15)	0.0268 (15)	0.0080 (12)	0.0080 (12)	0.0113 (12)
Cl24	0.0329 (4)	0.0368 (4)	0.0394 (4)	0.0123 (3)	0.0157 (3)	0.0182 (3)
C225	0.0339 (16)	0.0198 (14)	0.0290 (15)	0.0077 (12)	0.0059 (12)	0.0078 (11)
C226	0.0341 (16)	0.0193 (13)	0.0257 (15)	0.0120 (12)	0.0046 (12)	0.0043 (11)
N23	0.0304 (13)	0.0182 (11)	0.0208 (12)	0.0106 (10)	0.0065 (10)	0.0058 (9)
C24	0.0303 (15)	0.0186 (13)	0.0180 (13)	0.0101 (11)	0.0048 (11)	0.0031 (10)
C241	0.0299 (15)	0.0177 (13)	0.0271 (15)	0.0093 (11)	0.0080 (12)	0.0055 (11)
C242	0.0287 (15)	0.0206 (13)	0.0293 (15)	0.0102 (11)	0.0079 (12)	0.0081 (11)
C243	0.0291 (16)	0.0205 (14)	0.0392 (18)	0.0087 (12)	0.0054 (13)	0.0048 (12)
C244	0.0313 (16)	0.0236 (15)	0.049 (2)	0.0151 (13)	0.0103 (14)	−0.0058 (14)
C245	0.045 (2)	0.0314 (17)	0.0376 (18)	0.0164 (15)	0.0217 (15)	0.0055 (14)
C246	0.0420 (18)	0.0286 (15)	0.0254 (15)	0.0191 (14)	0.0118 (13)	0.0056 (12)
C25	0.0275 (14)	0.0186 (13)	0.0203 (13)	0.0090 (11)	0.0047 (11)	0.0017 (10)
C251	0.0308 (15)	0.0201 (13)	0.0224 (14)	0.0132 (11)	0.0076 (11)	0.0062 (11)
C252	0.0346 (16)	0.0252 (14)	0.0252 (15)	0.0122 (13)	0.0068 (12)	0.0042 (12)
C253	0.0353 (17)	0.0254 (15)	0.0439 (19)	0.0091 (13)	0.0164 (15)	0.0075 (14)
C254	0.0447 (19)	0.0304 (16)	0.0388 (18)	0.0208 (15)	0.0231 (15)	0.0181 (14)
C255	0.0454 (19)	0.0328 (16)	0.0238 (15)	0.0238 (15)	0.0112 (13)	0.0127 (13)
C256	0.0370 (17)	0.0269 (14)	0.0208 (14)	0.0171 (13)	0.0082 (12)	0.0051 (11)

### Geometric parameters (Å, º)

**Table:**

N11—C12	1.368 (4)	N21—C22	1.365 (4)
N11—C15	1.388 (4)	N21—C25	1.388 (4)
N11—C111	1.469 (4)	N21—C211	1.468 (4)
C111—C112	1.525 (4)	C211—C212	1.530 (4)
C111—H11A	0.9900	C211—H21A	0.9900
C111—H11B	0.9900	C211—H21B	0.9900
C112—O112	1.413 (3)	C212—O212	1.408 (4)
C112—C113	1.524 (4)	C212—C213	1.525 (4)
C112—H112	1.0000	C212—H212	1.0000
O112—H12O	0.80 (5)	O212—H22O	0.82 (5)
C113—H11C	0.9800	C213—H21C	0.9800
C113—H11D	0.9800	C213—H21D	0.9800
C113—H11E	0.9800	C213—H21E	0.9800
C12—N13	1.330 (4)	C22—N23	1.330 (4)
C12—C121	1.477 (4)	C22—C221	1.475 (4)
C121—C126	1.399 (4)	C221—C226	1.396 (4)
C121—C122	1.402 (4)	C221—C222	1.402 (4)
C122—C123	1.383 (4)	C222—C223	1.390 (4)
C122—H122	0.9500	C222—H222	0.9500
C123—C124	1.384 (5)	C223—C224	1.378 (4)
C123—H123	0.9500	C223—H223	0.9500
C124—C125	1.386 (5)	C224—C225	1.387 (5)
C124—Cl14	1.743 (3)	C224—Cl24	1.747 (3)
C125—C126	1.391 (4)	C225—C226	1.383 (4)
C125—H125	0.9500	C225—H225	0.9500
C126—H126	0.9500	C226—H226	0.9500
N13—C14	1.373 (4)	N23—C24	1.377 (4)
C14—C15	1.376 (4)	C24—C25	1.370 (4)
C14—C141	1.476 (4)	C24—C241	1.471 (4)
C141—C146	1.394 (4)	C241—C242	1.398 (4)
C141—C142	1.395 (4)	C241—C246	1.398 (4)
C142—C143	1.393 (4)	C242—C243	1.384 (4)
C142—H142	0.9500	C242—H242	0.9500
C143—C144	1.395 (5)	C243—C244	1.403 (5)
C143—H143	0.9500	C243—H243	0.9500
C144—C145	1.396 (5)	C244—C245	1.376 (5)
C144—H144	0.9500	C244—H244	0.9500
C145—C146	1.391 (4)	C245—C246	1.396 (5)
C145—H145	0.9500	C245—H245	0.9500
C146—H146	0.9500	C246—H246	0.9500
C15—C151	1.480 (4)	C25—C251	1.478 (4)
C151—C156	1.397 (4)	C251—C252	1.390 (5)
C151—C152	1.401 (4)	C251—C256	1.401 (4)
C152—C153	1.391 (4)	C252—C253	1.387 (4)
C152—H152	0.9500	C252—H252	0.9500
C153—C154	1.382 (5)	C253—C254	1.395 (5)
C153—H153	0.9500	C253—H253	0.9500
C154—C155	1.386 (5)	C254—C255	1.387 (5)
C154—H154	0.9500	C254—H254	0.9500
C155—C156	1.392 (4)	C255—C256	1.388 (4)
C155—H155	0.9500	C255—H255	0.9500
C156—H156	0.9500	C256—H256	0.9500
			
C12—N11—C15	106.9 (2)	C22—N21—C25	107.0 (2)
C12—N11—C111	129.2 (2)	C22—N21—C211	129.2 (3)
C15—N11—C111	123.9 (2)	C25—N21—C211	123.6 (2)
N11—C111—C112	112.1 (2)	N21—C211—C212	112.9 (2)
N11—C111—H11A	109.2	N21—C211—H21A	109.0
C112—C111—H11A	109.2	C212—C211—H21A	109.0
N11—C111—H11B	109.2	N21—C211—H21B	109.0
C112—C111—H11B	109.2	C212—C211—H21B	109.0
H11A—C111—H11B	107.9	H21A—C211—H21B	107.8
O112—C112—C113	112.5 (2)	O212—C212—C213	112.7 (3)
O112—C112—C111	106.4 (2)	O212—C212—C211	106.5 (2)
C113—C112—C111	110.6 (3)	C213—C212—C211	110.5 (3)
O112—C112—H112	109.1	O212—C212—H212	109.0
C113—C112—H112	109.1	C213—C212—H212	109.0
C111—C112—H112	109.1	C211—C212—H212	109.0
C112—O112—H12O	113 (3)	C212—O212—H22O	111 (3)
C112—C113—H11C	109.5	C212—C213—H21C	109.5
C112—C113—H11D	109.5	C212—C213—H21D	109.5
H11C—C113—H11D	109.5	H21C—C213—H21D	109.5
C112—C113—H11E	109.5	C212—C213—H21E	109.5
H11C—C113—H11E	109.5	H21C—C213—H21E	109.5
H11D—C113—H11E	109.5	H21D—C213—H21E	109.5
N13—C12—N11	111.0 (2)	N23—C22—N21	110.8 (3)
N13—C12—C121	121.5 (3)	N23—C22—C221	121.3 (2)
N11—C12—C121	127.3 (3)	N21—C22—C221	127.9 (3)
C126—C121—C122	118.7 (3)	C226—C221—C222	118.8 (3)
C126—C121—C12	124.1 (3)	C226—C221—C22	118.7 (3)
C122—C121—C12	117.0 (3)	C222—C221—C22	122.3 (3)
C123—C122—C121	121.1 (3)	C223—C222—C221	119.9 (3)
C123—C122—H122	119.5	C223—C222—H222	120.0
C121—C122—H122	119.5	C221—C222—H222	120.0
C122—C123—C124	119.0 (3)	C224—C223—C222	119.8 (3)
C122—C123—H123	120.5	C224—C223—H223	120.1
C124—C123—H123	120.5	C222—C223—H223	120.1
C123—C124—C125	121.4 (3)	C223—C224—C225	121.4 (3)
C123—C124—Cl14	119.2 (3)	C223—C224—Cl24	119.3 (3)
C125—C124—Cl14	119.4 (2)	C225—C224—Cl24	119.3 (2)
C124—C125—C126	119.3 (3)	C226—C225—C224	118.7 (3)
C124—C125—H125	120.3	C226—C225—H225	120.7
C126—C125—H125	120.3	C224—C225—H225	120.7
C125—C126—C121	120.4 (3)	C225—C226—C221	121.3 (3)
C125—C126—H126	119.8	C225—C226—H226	119.3
C121—C126—H126	119.8	C221—C226—H226	119.3
C12—N13—C14	106.3 (2)	C22—N23—C24	106.4 (2)
N13—C14—C15	109.8 (3)	C25—C24—N23	109.7 (3)
N13—C14—C141	122.7 (3)	C25—C24—C241	127.9 (3)
C15—C14—C141	127.5 (3)	N23—C24—C241	122.5 (2)
C146—C141—C142	118.9 (3)	C242—C241—C246	118.4 (3)
C146—C141—C14	121.5 (3)	C242—C241—C24	120.9 (3)
C142—C141—C14	119.6 (3)	C246—C241—C24	120.7 (3)
C143—C142—C141	120.9 (3)	C243—C242—C241	120.8 (3)
C143—C142—H142	119.6	C243—C242—H242	119.6
C141—C142—H142	119.6	C241—C242—H242	119.6
C142—C143—C144	120.0 (3)	C242—C243—C244	120.3 (3)
C142—C143—H143	120.0	C242—C243—H243	119.8
C144—C143—H143	120.0	C244—C243—H243	119.8
C143—C144—C145	119.3 (3)	C245—C244—C243	119.1 (3)
C143—C144—H144	120.3	C245—C244—H244	120.4
C145—C144—H144	120.3	C243—C244—H244	120.4
C146—C145—C144	120.4 (3)	C244—C245—C246	120.7 (3)
C146—C145—H145	119.8	C244—C245—H245	119.7
C144—C145—H145	119.8	C246—C245—H245	119.7
C145—C146—C141	120.5 (3)	C245—C246—C241	120.6 (3)
C145—C146—H146	119.7	C245—C246—H246	119.7
C141—C146—H146	119.7	C241—C246—H246	119.7
C14—C15—N11	106.0 (2)	C24—C25—N21	106.1 (2)
C14—C15—C151	129.1 (3)	C24—C25—C251	128.5 (3)
N11—C15—C151	124.9 (3)	N21—C25—C251	125.3 (3)
C156—C151—C152	119.5 (3)	C252—C251—C256	119.1 (3)
C156—C151—C15	118.6 (3)	C252—C251—C25	119.0 (3)
C152—C151—C15	121.8 (3)	C256—C251—C25	121.8 (3)
C153—C152—C151	119.6 (3)	C253—C252—C251	121.0 (3)
C153—C152—H152	120.2	C253—C252—H252	119.5
C151—C152—H152	120.2	C251—C252—H252	119.5
C154—C153—C152	120.7 (3)	C252—C253—C254	119.6 (3)
C154—C153—H153	119.7	C252—C253—H253	120.2
C152—C153—H153	119.7	C254—C253—H253	120.2
C153—C154—C155	120.0 (3)	C255—C254—C253	119.8 (3)
C153—C154—H154	120.0	C255—C254—H254	120.1
C155—C154—H154	120.0	C253—C254—H254	120.1
C154—C155—C156	120.2 (3)	C254—C255—C256	120.5 (3)
C154—C155—H155	119.9	C254—C255—H255	119.7
C156—C155—H155	119.9	C256—C255—H255	119.7
C155—C156—C151	120.1 (3)	C255—C256—C251	120.0 (3)
C155—C156—H156	120.0	C255—C256—H256	120.0
C151—C156—H156	120.0	C251—C256—H256	120.0
			
C12—N11—C111—C112	−107.3 (3)	C22—N21—C211—C212	111.7 (3)
C15—N11—C111—C112	72.6 (3)	C25—N21—C211—C212	−72.8 (3)
N11—C111—C112—O112	64.4 (3)	N21—C211—C212—O212	−64.4 (3)
N11—C111—C112—C113	−173.2 (2)	N21—C211—C212—C213	172.9 (3)
C15—N11—C12—N13	1.1 (3)	C25—N21—C22—N23	−0.9 (3)
C111—N11—C12—N13	−179.0 (3)	C211—N21—C22—N23	175.1 (3)
C15—N11—C12—C121	176.0 (3)	C25—N21—C22—C221	178.1 (3)
C111—N11—C12—C121	−4.1 (5)	C211—N21—C22—C221	−5.9 (5)
N13—C12—C121—C126	−140.0 (3)	N23—C22—C221—C226	−36.8 (4)
N11—C12—C121—C126	45.6 (5)	N21—C22—C221—C226	144.3 (3)
N13—C12—C121—C122	37.1 (4)	N23—C22—C221—C222	138.1 (3)
N11—C12—C121—C122	−137.3 (3)	N21—C22—C221—C222	−40.7 (5)
C126—C121—C122—C123	−0.5 (5)	C226—C221—C222—C223	1.0 (4)
C12—C121—C122—C123	−177.7 (3)	C22—C221—C222—C223	−174.0 (3)
C121—C122—C123—C124	0.5 (5)	C221—C222—C223—C224	−0.5 (5)
C122—C123—C124—C125	−0.2 (5)	C222—C223—C224—C225	−0.3 (5)
C122—C123—C124—Cl14	178.1 (3)	C222—C223—C224—Cl24	178.2 (2)
C123—C124—C125—C126	−0.2 (5)	C223—C224—C225—C226	0.5 (5)
Cl14—C124—C125—C126	−178.5 (3)	Cl24—C224—C225—C226	−178.0 (2)
C124—C125—C126—C121	0.2 (5)	C224—C225—C226—C221	0.1 (5)
C122—C121—C126—C125	0.1 (5)	C222—C221—C226—C225	−0.8 (5)
C12—C121—C126—C125	177.1 (3)	C22—C221—C226—C225	174.3 (3)
N11—C12—N13—C14	−0.6 (3)	N21—C22—N23—C24	0.3 (3)
C121—C12—N13—C14	−175.9 (3)	C221—C22—N23—C24	−178.8 (3)
C12—N13—C14—C15	0.0 (3)	C22—N23—C24—C25	0.5 (3)
C12—N13—C14—C141	179.2 (3)	C22—N23—C24—C241	179.0 (3)
N13—C14—C141—C146	44.4 (4)	C25—C24—C241—C242	143.7 (3)
C15—C14—C141—C146	−136.5 (3)	N23—C24—C241—C242	−34.6 (4)
N13—C14—C141—C142	−136.9 (3)	C25—C24—C241—C246	−35.6 (5)
C15—C14—C141—C142	42.2 (4)	N23—C24—C241—C246	146.1 (3)
C146—C141—C142—C143	0.3 (4)	C246—C241—C242—C243	1.2 (5)
C14—C141—C142—C143	−178.4 (3)	C24—C241—C242—C243	−178.2 (3)
C141—C142—C143—C144	0.9 (5)	C241—C242—C243—C244	0.8 (5)
C142—C143—C144—C145	−1.3 (5)	C242—C243—C244—C245	−2.0 (5)
C143—C144—C145—C146	0.6 (5)	C243—C244—C245—C246	1.3 (5)
C144—C145—C146—C141	0.6 (5)	C244—C245—C246—C241	0.7 (5)
C142—C141—C146—C145	−1.0 (4)	C242—C241—C246—C245	−1.9 (5)
C14—C141—C146—C145	177.7 (3)	C24—C241—C246—C245	177.4 (3)
N13—C14—C15—N11	0.7 (3)	N23—C24—C25—N21	−1.0 (3)
C141—C14—C15—N11	−178.6 (3)	C241—C24—C25—N21	−179.5 (3)
N13—C14—C15—C151	−177.3 (3)	N23—C24—C25—C251	174.7 (3)
C141—C14—C15—C151	3.5 (5)	C241—C24—C25—C251	−3.8 (5)
C12—N11—C15—C14	−1.0 (3)	C22—N21—C25—C24	1.1 (3)
C111—N11—C15—C14	179.0 (2)	C211—N21—C25—C24	−175.2 (3)
C12—N11—C15—C151	177.0 (3)	C22—N21—C25—C251	−174.7 (3)
C111—N11—C15—C151	−2.9 (4)	C211—N21—C25—C251	9.0 (4)
C14—C15—C151—C156	56.7 (4)	C24—C25—C251—C252	−59.1 (4)
N11—C15—C151—C156	−120.9 (3)	N21—C25—C251—C252	115.8 (3)
C14—C15—C151—C152	−119.2 (3)	C24—C25—C251—C256	117.0 (4)
N11—C15—C151—C152	63.2 (4)	N21—C25—C251—C256	−68.1 (4)
C156—C151—C152—C153	0.7 (4)	C256—C251—C252—C253	−0.7 (5)
C15—C151—C152—C153	176.5 (3)	C25—C251—C252—C253	175.4 (3)
C151—C152—C153—C154	−0.4 (4)	C251—C252—C253—C254	0.0 (5)
C152—C153—C154—C155	−0.2 (5)	C252—C253—C254—C255	0.6 (5)
C153—C154—C155—C156	0.4 (5)	C253—C254—C255—C256	−0.6 (5)
C154—C155—C156—C151	−0.1 (5)	C254—C255—C256—C251	−0.2 (5)
C152—C151—C156—C155	−0.4 (4)	C252—C251—C256—C255	0.8 (4)
C15—C151—C156—C155	−176.4 (3)	C25—C251—C256—C255	−175.3 (3)

### Hydrogen-bond geometry (Å, º)

Cg1, Cg2, Cg5 and Cg6 are the centroids of the N11/C12/N13/C14/C15, N21/C22/N23/C24/C25, C221–C226 and C241–C246 rings, respectively.

**Table:**

*D*—H···*A*	*D*—H	H···*A*	*D*···*A*	*D*—H···*A*
O112—H12*O*···N23^i^	0.80 (5)	2.01 (5)	2.804 (3)	170 (4)
O212—H22*O*···N13^ii^	0.82 (5)	1.99 (5)	2.790 (3)	165 (4)
C152—H152···O212^iii^	0.95	2.61	3.227 (4)	123
C256—H256···O112	0.95	2.48	3.162 (4)	129
C242—H242···O112^i^	0.95	2.68	3.277 (4)	122
C243—H243···Cl24^iv^	0.95	2.91	3.836 (3)	166
C113—H11*D*···*Cg*6^iii^	0.98	2.96	3.778 (3)	142
C153—H153···*Cg*5^iii^	0.95	2.65	3.495 (3)	148
C213—H21*D*···*Cg*2	0.98	2.91	3.745 (4)	144
C255—H255···*Cg*1	0.95	2.65	3.505 (3)	149

Symmetry codes: (i) −*x*+1, −*y*+1, −*z*+1; (ii) −*x*+1, −*y*+2, −*z*+1; (iii) *x*, *y*, *z*−1; (iv) *x*+1, *y*, *z*.

## References

[bb1] Agilent (2014). *CrysAlis PRO*. Agilent Technologies, Yarnton, England.

[bb2] Akkurt, M., Jasinski, J. P., Mohamed, S. K., Marzouk, A. A. & Albayati, M. R. (2015). *Acta Cryst.* E**71**, o299–o300. 10.1107/S2056989015006763 PMC4420112 25995914

[bb3] Allen, F. H., Johnson, O., Shields, G. P., Smith, B. R. & Towler, M. (2004). *J. Appl. Cryst.* **37**, 335–338.

[bb4] Bahnous, M., Bouraiou, A., Chelghoum, M., Bouacida, S., Roisnel, T., Smati, F., Bentchouala, C., Gros, P. C. & Belfaitah, A. (2013). *Bioorg. Med. Chem. Lett.* **23**, 1274–1278. 10.1016/j.bmcl.2013.01.004 23374871

[bb5] Belwal, C. & Joshi, A. (2012). *Der Pharma Chem.* **4**, 1873–1878.

[bb7] Farrugia, L. J. (2012). *J. Appl. Cryst.* **45**, 849–854.

[bb8] Ghoranneviss, M., Mohammadi Ziarani, G., Abbasi, A., Hantehzadeh, M. R. & Farahani, Z. (2008). *Acta Cryst.* E**64**, o1233. 10.1107/S1600536808016802 PMC2961831 21202870

[bb9] Groom, C. R., Bruno, I. J., Lightfoot, M. P. & Ward, S. C. (2016). *Acta Cryst.* B**72**, 171–179. 10.1107/S2052520616003954 PMC4822653 27048719

[bb10] Hunter, K. A. & Simpson, J. (1999). *TITAN2000*. University of Otago, New Zealand.

[bb11] Jasinski, J. P., Mohamed, S. K., Akkurt, M., Abdelhamid, A. A. & Albayati, M. R. (2015). *Acta Cryst.* E**71**, o77–o78. 10.1107/S2056989014027078 PMC4384621 25878880

[bb12] Kapoor, K., Gupta, V. K., Rajnikant, Gupta, P. & Paul, S. (2011). *X-ray Str. Anal. Online*, **27**, 53–54.

[bb13] Macrae, C. F., Bruno, I. J., Chisholm, J. A., Edgington, P. R., McCabe, P., Pidcock, E., Rodriguez-Monge, L., Taylor, R., van de Streek, J. & Wood, P. A. (2008). *J. Appl. Cryst.* **41**, 466–470.

[bb14] Marzouk, A. A., Abdelhamid, A. A., Mohamed, S. K. & Simpson, J. (2016). *Z. Naturforsch. Teil B*. 10.1515/znb-2016-0121.

[bb15] Mohamed, S. K., Akkurt, M., Marzouk, A. A., Abbasov, V. M. & Gurbanov, A. V. (2013*a*). *Acta Cryst.* E**69**, o474–o475. 10.1107/S1600536813004285 PMC3629514 23634032

[bb16] Mohamed, S. K., Akkurt, M., Marzouk, A. A. E., Santoyo-Gonzalez, F. & Elremaily, M. A. A. (2013*c*). *Acta Cryst.* E**69**, o875–o876. 10.1107/S1600536813012592 PMC3685034 23795053

[bb17] Mohamed, S. K., Akkurt, M., Singh, K., Marzouk, A. A. & Abdelhamid, A. A. (2013*b*). *Acta Cryst.* E**69**, o1243. 10.1107/S1600536813018229 PMC3793746 24109333

[bb18] Mohamed, S. K., Simpson, J., Marzouk, A. A., Talybov, A. H., Abdelhamid, A. A., Abdullayev, Y. A. & Abbasov, V. M. (2015). *Z. Naturforsch. Teil B*, **70**, 809–817.

[bb19] Mohammadi, A., Keshvari, H., Sandaroos, R., Maleki, B., Rouhi, H., Moradi, H., Sepehr, Z. & Damavandi, S. (2012). *App. Catalysis A General*, **37**, 429–430.

[bb20] Rajaraman, D., Sundararajan, G., Rajkumar, R., Bharanidharan, S. & Krishnasamy, K. (2016). *J. Mol. Struct.* **1108**, 698–707.

[bb21] Sharma, D., Narasimhan, B., Kumar, P., Judge, V., Narang, R., De Clercq, E. & Balzarini, J. (2009). *Eur. J. Med. Chem.* **44**, 2347–2353. 10.1016/j.ejmech.2008.08.010 18851889

[bb22] Sheldrick, G. M. (2008). *Acta Cryst.* A**64**, 112–122. 10.1107/S0108767307043930 18156677

[bb23] Sheldrick, G. M. (2015). *Acta Cryst.* C**71**, 3–8.

[bb24] Spek, A. L. (2009). *Acta Cryst.* D**65**, 148–155. 10.1107/S090744490804362X PMC2631630 19171970

[bb25] Sridharan, S., Sabarinathan, N. & Antony, S. A. (2014). *Int. J. Chem. Tech. Res.* **6**, 1220–1227.

[bb26] Westrip, S. P. (2010). *J. Appl. Cryst.* **43**, 920–925.

[bb27] Yanover, D. & Kaftory, M. (2009). *Acta Cryst.* E**65**, o711. 10.1107/S1600536809006552 PMC2968781 21582448

